# Clinical outcomes of pembrolizumab plus gemcitabine and cisplatin in a Japanese institutional subgroup of KEYNOTE-966: a dexamethasone-minimizing supportive-care approach

**DOI:** 10.3389/fonc.2026.1806667

**Published:** 2026-09-21

**Authors:** Satoshi Kobayashi, Tomomi Hamaguchi, Ritsuko Oishi, Yuichiro Tozuka, Taito Fukushima, Kouji Yamamoto, Junji Furuse, Makoto Ueno

**Affiliations:** 1 Department of Gastroenterology, Kanagawa Cancer Center, Yokohama, Japan; 2 Department of Biostatistics, Yokohama City University School of Medicine, Yokohama, Japan

**Keywords:** antibiotic, biliary tract cancer, dexamethasone, immune checkpoint inhibitors, pembrolizumab, steroid

## Abstract

**Introduction:**

KEYNOTE-966, a double-blinded randomized controlled phase III trial comparing pembrolizumab/placebo + gemcitabine + cisplatin (GC) for biliary tract cancer [BTC], demonstrated the efficacy and safety of addition of pembrolizumab to GC. The effect of steroid and antibiotics use on immune check-point inhibitors is still of interest.

**Methods:**

This was a *post-hoc*, exploratory institutional subgroup analysis of KEYNOTE-966. Dexamethasone was minimized for antiemetic prophylaxis, and antibiotics were administered selectively for cholangitis according to the institutional practice. Thirty-eight patients registered at our institution were randomly assigned to each treatment group. After the key was opened following completion of the protocol treatment in these patients, we compared the efficacy and safety in patients of Pembrolizumab+GC and those of Placebo+GC. We performed multivariable analysis using the Cox regression hazard model and inverse-probability weighting propensity score (IPW) analysis to adjust for between-group background differences.

**Results:**

It was found that there were 22 and 16 patients in the Pembrolizumab+GC and Placebo+GC groups, respectively. The hazard ratios (HRs) of overall survival (OS) and progression-free survival (PFS) for the Pembrolizumab+GC group were 0.622 (95% confidence interval [CI], 0.318–1.217) and 0.360 (95% CI, 0.178–0.728), respectively; the objective response rates were 31.8% and 6.3%, respectively. The HR for OS and PFS in the multivariable and IPW analyses were numerically similar to those in the original analysis, which was better than that observed in the entire KEYNOTE-966 cohort.

**Discussion:**

Our experience of Pembrolizumab + GC over Placebo +GC generates a clinical question whether the efficacy of Pembrolizumab may be enhanced when antibiotics and antiemetic DEX are avoided.

## 1 Introduction

Biliary tract cancer (BTC) has a poor prognosis because most patients are diagnosed at an unresectable stage, and surgical resection is the only curative treatment available. Palliative systemic therapy is the standard treatment for patients with unresectable diseases (1). Gemcitabine (Gem) plus Cisplatin (CDDP) had been a standard therapy until 2022 (2). However, over the past several years, based on the two global phase III studies, the TOPAZ-1 study (3) and KEYNOTE-966 study (4), immune checkpoint inhibitors (ICIs) have emerged as a clinical daily practice for the treatment of patients with unresectable BTCs. Durvalumab plus Gem+CDDP and pembrolizumab plus Gem+CDDP have shown superior overall survival (OS) to placebo+Gem+CDDP as a first-line therapy for patients with unresectable or recurrent BTC.

The impact of the combination of ICIs with cytotoxic agents on survival was not remarkable in these trials compared with that for other cancers, with hazard ratios of 0.80 or 0.83 (3, 4). One reason for this might be that BTC has a stroma rich in fibrous components, making it difficult for immune cells to infiltrate compared with other cancers (5). Furthermore, BTC often coexists with biliary tract infections and requires frequent administration of antibiotics. Antibiotic use decreases the efficacy of ICIs by changing the microbiome and decreasing its abundance (6–8). Corticosteroids are also of concern as concomitant medications because they may affect the efficacy of ICIs. Dexamethasone (DEX) at a dose of 8–12 mg/day is administered on days 1 and 8 at minimum, and days 1–3 and days 8–10 at maximum within 21 days of the cycle in the pembrolizumab+Gem+CDDP regimen because CDDP is classified as a high risk for chemotherapy-induced nausea and vomiting, even at a low dose of 25 mg/m^2^ (9). Patients treated with prednisone at the initiation of ICI treatment had poor survival (10, 11); therefore, we anticipated that the antiemetic use of DEX might worsen the efficacy of pembrolizumab+Gem+CDDP in patients with unresectable or recurrent BTCs. However, because the corticosteroids tested in earlier reports were used for symptom relief and baseline use, it was unclear whether their use contributed to treatment efficacy (12, 13). Considering this background, we attempted to minimize prophylactic DEX by using fosnetupitant/palonosetron and adopted an antibiotic-sparing approach for cholangitis when clinically appropriate to patients participating in the KEYNOTE-966 trial.

However, because these supportive-care practices were applied to patients in both randomized treatment groups, this institutional subgroup analysis was not designed to directly estimate the causal effects of DEX or antibiotic avoidance. The present study therefore aimed to describe the efficacy and safety of pembrolizumab plus Gem+CDDP compared with placebo plus Gem+CDDP in patients enrolled to KEYONTE-966 at our institution and to characterize the supportive-care practices used during treatment.

## 2 Materials and methods

### 2.1 Patients

This was a *post-hoc*, exploratory analysis of patients enrolled at Kanagawa Cancer Center in the KEYNOTE-966 randomized controlled trial.

A total of 38 patients at our institution were enrolled in the KEYNOTE-966 trial (ClinicalTrials.gov, NCT04003636). The eligibility criteria for the KEYNOTE-966 trial have been described in previous reports (4). Briefly, patients were required to have an Eastern Cooperative oncology Group performance status (ECOG PS) of 0 or 1, be pathologically diagnosed with adenocarcinoma, have locally advanced or distant metastasis, have an expected survival of over three months, and be untreated for unresectable or recurrent BTC. The patients were randomly assigned to the Pembrolizumab and Placebo groups in a 1:1 ratio. The KEYNOTE-966 trial was conducted in a double-blind manner, with neither the patients nor their attending physicians being aware of the group allocation.

### 2.2 Treatment

The administration of placebo+Gem+CDDP or pembrolizumab+Gem+CDDP followed the protocol described in published reports. Briefly, 1000 mg/m² Gem and 25 mg/m² CDDP were administered on days 1 and 8, repeated every three weeks. The CDDP administration was limited to a maximum of 24 cycles. Placebo or pembrolizumab was administered on day 1 and repeated every three weeks for up to 2 years. Treatment was continued until disease progression, drug intolerance, or patient refusal occurred.

### 2.3 Supportive care approaches

For the prevention of nausea and vomiting caused by Gem and CDDP, various guidelines recommend administering DEX 9.9–13.2 mg and a 5-hydroxytryptamine 3 receptor antagonist (5-HT_3_RA) on day 1, and oral DEX 4–8 mg on days 2–3. At our institution, we avoided DEX by administering a drip infusion of 235 mg of fosnetupitant and 0.75 mg of palonosetron before administering Gem and CDDP. If nausea or vomiting occurred during treatment, antiemetics such as domperidone or metoclopramide were administered orally, and if no improvement was achieved, DEX was used. Prednisolone or hydrocortisone administration for immune-related adverse events (irAEs) was not restricted. If fever or elevated hepatobiliary enzymes were observed during the treatment course, leading to a diagnosis of cholangitis, biliary drainage was performed within 24 h of diagnosis. Nasobiliary drainage was primarily used to ensure effective biliary drainage. Antibiotics were administered only if fever did not resolve or vital signs did not normalize with biliary drainage alone. However, if antibiotic administration was deemed necessary, it was discontinued as soon as vital signs, including body temperature, stabilized. When bile juice from the drainage tube became clear and no debris was observed, the nasobiliary tube was removed and replaced with a biliary stent. Plastic or metal stents of sufficient length to cover the biliary strictures were selected for use.

### 2.4 Treatment outcomes

Fisher’s exact test and the Wilcoxon rank-sum test (Mann–Whitney U test) were used to compare categorical and continuous variables between the treatment groups, respectively. Overall survival was defined as the period from the start of each treatment protocol to the date of death or the last confirmation of survival. Progression-free survival was defined as the period from the start of each treatment protocol to an earlier date of disease progression or death. Adverse events and radiological responses were evaluated according to the Common Terminology Criteria for Adverse Events version 5.0 and Response Evaluation Criteria in Solid Tumors version 1.1, as defined in the KEYNOTE-966 protocol (4).

### 2.5 Statistical analysis

For patient characteristics in each treatment group, continuous variables are expressed as medians with interquartile ranges (IQRs), and categorical values are expressed as frequencies with percentages. Time-to-event values were calculated using the Kaplan–Meier method to determine the median value with a 95% confidence interval (CI), and comparisons between two groups were performed using the log-rank test. In addition, multivariable analysis using the Cox regression hazard model and the inverse probability weighting propensity score (IPW) analysis were performed to adjust for differences in patient backgrounds. For the Cox regression hazard model, we used ECOG PS (0 vs. 1), disease status (locally advanced vs. metastatic), CA 19-9, and treatment group as covariates. CA 19–9 was treated as a continuous variable and transformed as ln(CA 19-9 + 1) to account for its markedly right-skewed distribution and zero values. Non-linearity in the association between log-transformed CA 19–9 and each survival outcome was assessed by comparing models with linear and restricted cubic spline terms. Given evidence of non-linearity, CA 19–9 was modeled using a restricted cubic spline with three knots placed at the 10th, 50th, and 90th percentiles of its distribution. In addition to these four factors, age, sex (male vs. female), primary location (gallbladder vs. others), CRP, albumin, NLR were used as the covariates for IPW analysis. Standardized mean differences (SMDs) were calculated for each patient background, and we assumed that the backgrounds of the treatment groups in the IPW analysis were well balanced when the SMD was <0.2. All statistical analyses were performed using SAS, version 9.4 (SAS Institute, Cary, NC, USA).

### 2.6 Ethics statement and data governance

The present analysis was not prespecified in the KEYNOTE-966 protocol. Written informed consent was obtained for secondary use of the data in all the patients. Additionally, all the patients were given the choice to opt out of having their information used in the study. After the data from the KEYNOTE-966 trial were locked for the primary endpoint, the key was opened with sponsor authorization. The publication of this study was also permitted by the sponsor.

## 3 Results

### 3.1 Patients

Twenty-two and 16 patients were assigned to the pembrolizumab and placebo groups, respectively. The patient characteristics for each treatment group are shown in **Table 1**. There was no missing data, and the data shown in this table was from complete case. There were some differences in the distribution of patient backgrounds between the groups that may be considered prognostic factors for survival in advanced BTCs: albumin and neutrophil–lymphocyte ratios (NLRs), Log (CA 19-9 + 1), gallbladder carcinoma, an ECOG PS of 0, and metastatic disease with SMD >0.2, even though patients were randomly assigned to each treatment arm. Among the Pembrolizumab and Placebo groups, nine (40.9%) and four (25.0%) patients had undergone biliary stent placement, and 13 (59.1%) and five (31.3%) patients had received antibiotics within 1 month of study registration, respectively. None of the patients received corticosteroids at the time of treatment initiation.

**Table 1:** Patient characteristics.

N (%)	Pembrolizumab+GC N=22	Placebo+GC N=16	SMD
Age (years) Median (IQR)	70.5 (65.5–73.0)	71 (63.0–77.0)	0.026
Sex: Male	12 (55)	8 (50)	0.091
ECOG PS: 0	18 (82)	10 (63)	0.441
Site of origin Distal bile duct Hilar bile duct Intrahepatic bile duct Gallbladder	5 (23) 2 (9) 12 (55) 3 (14)	1 (6) 2 (13) 8 (50) 5 (31)	0.432
Disease status Locally advanced Metastatic	10 (45) 12 (55)	3 (19) 13 (81)	0.597
CRP (mg/dL) Median (IQR)	1.39 (0.18–1.76)	1.06 (0.45–1.72)	0.062
Albumin (g/dL) Median (IQR)	3.9 (3.7–4.0)	3.7 (3.5–3.9)	0.332
NLR Median (IQR)	2.76 (1.68–4.03)	2.94 (2.16–5.56)	0.381
CA 19-9 (U/mL) Median (IQR)	91.0 (20.2–336.1)	905.1 (22.3–11037.8)	0.408^†^
Biliary stent Present	9 (41)	4 (25)	
Antibiotic use within 1 month of study start	13 (59)	5 (31)	

GC, gemcitabine + cisplatin; SMD, standardized mean difference; IQR, interquartile range; CRP, C-reactive protein; NLR, neutrophil–lymphocyte ratio; CA 19-9, carbohydrate antigen 19-9.

† For CA 19-9, the standardized mean difference was calculated using ln(CA 19-9 + 1) because of its markedly right-skewed distribution.

### 3.2 Supportive-care practices for infectious and emetic events

Regarding infectious events, nine patients experienced cholangitis and one experienced bacterial pneumonia in the pembrolizumab group (one had overlapping infections), whereas four patients experienced cholangitis in the placebo group. All of these infectious events were judged not to be related to protocol treatments, and these patients recovered with antibiotics and/or biliary drainage; two patients in the pembrolizumab group required only biliary drainage for the treatment of cholangitis. Antibiotics were administered to seven (32%) and four (25%) patients in the pembrolizumab and placebo groups, respectively (**Supplementary Table 1**). The median duration of antibiotic use was 4 days (range, 1–28) for each infectious event. The antibiotics used in each event were determined empirically and, based on the culture results of bile juice and/or blood, Ceftriaxone, Meropenem, Piperacillin/Tazobactam, and Ceftazidime were administered to eight, three, two, and two patients, respectively; Ampicillin/Sulbactam, Cefepime, and Amoxicillin–clavulanate were administered to one patient each.

All grades of nausea and vomiting were observed in nine (40.9%) and three (13.6%) patients in the pembrolizumab group, and seven (43.8%) and two (12.5%) patients in the placebo group, respectively. Consequently, domperidone or metoclopramide was administered to seven (31.8%) and seven patients (43.8%) in the pembrolizumab and placebo groups, respectively. None of the patients experienced grade 3 or higher nausea/vomiting or required the antiemetic steroids.

### 3.3 Immune-related adverse events

Six (27%) and one (6%) patient were diagnosed with irAEs in the pembrolizumab and placebo groups, respectively; interstitial lung disease, thrombocytopenia, and nausea in four, one, and one patient in the pembrolizumab group and liver dysfunction in one patient in the placebo group. All seven patients discontinued protocol treatment owing to these adverse events; four (18%) and one patient (6%) required systemic corticosteroids for the treatment of irAEs (**Supplementary Table 2**).

One patient in the pembrolizumab group received prednisolone for the treatment of sudden sensorineural hearing loss that was not considered an irAE. As a result, prednisolone was used for the treatment in five (23%) and one patient (6%) in the pembrolizumab and placebo groups, respectively.

### 3.4 Efficacy of pembrolizumab plus Gem+CDDP versus placebo plus Gem+CDDP

The reasons for discontinuation of the protocol treatment were progressive disease in 15 and 15 patients and adverse events in 6 and 1 patients in the pembrolizumab and placebo groups, respectively. Additionally, one patient completed the 2-year protocol treatment and is still alive without any treatment in the Pembrolizumab group. In the median follow-up of 14.9 months (range, 1.9–48.5) and 11.9 months (range, 1.7–56.2), we observed 21 and 16 events for PFS and 21 and 16 events for OS in the pembrolizumab and placebo groups, respectively. The median PFSs were 10.5 months (95% confidence interval [CI], 7.3–13.6) and 5.4 months (95% CI, 3.5–7.3) in the pembrolizumab and placebo groups, respectively (log-rank p=0.003, **Figure 1A**). The HR of PFS for the pembrolizumab group was 0.360 (95% CI, 0.178–0.728) compared with that of the placebo group. The median OS was 14.7 (95% confidence interval [CI], 9.9–19.4) months and 9.8 (95% CI, 9.2–10.3) months in the pembrolizumab and placebo groups, respectively (log-rank p=0.161, **Figure 1B**). The HR of OS for the pembrolizumab group was 0.622 (95% CI, 0.318–1.217) compared with that of the placebo group. The objective responses are listed in **Table 2**. A complete response was observed only in one patient in the pembrolizumab group. Among the pembrolizumab and placebo groups, a partial response was observed in 6 and 1 patients, and stable disease in 11 and 11 patients, respectively. The objective response rates were 31.8% and 6.3% (p=0.062), and the disease control rates were 81.8% and 75.0% (p=0.698) in the pembrolizumab and placebo groups, respectively.

**Figure 1:**
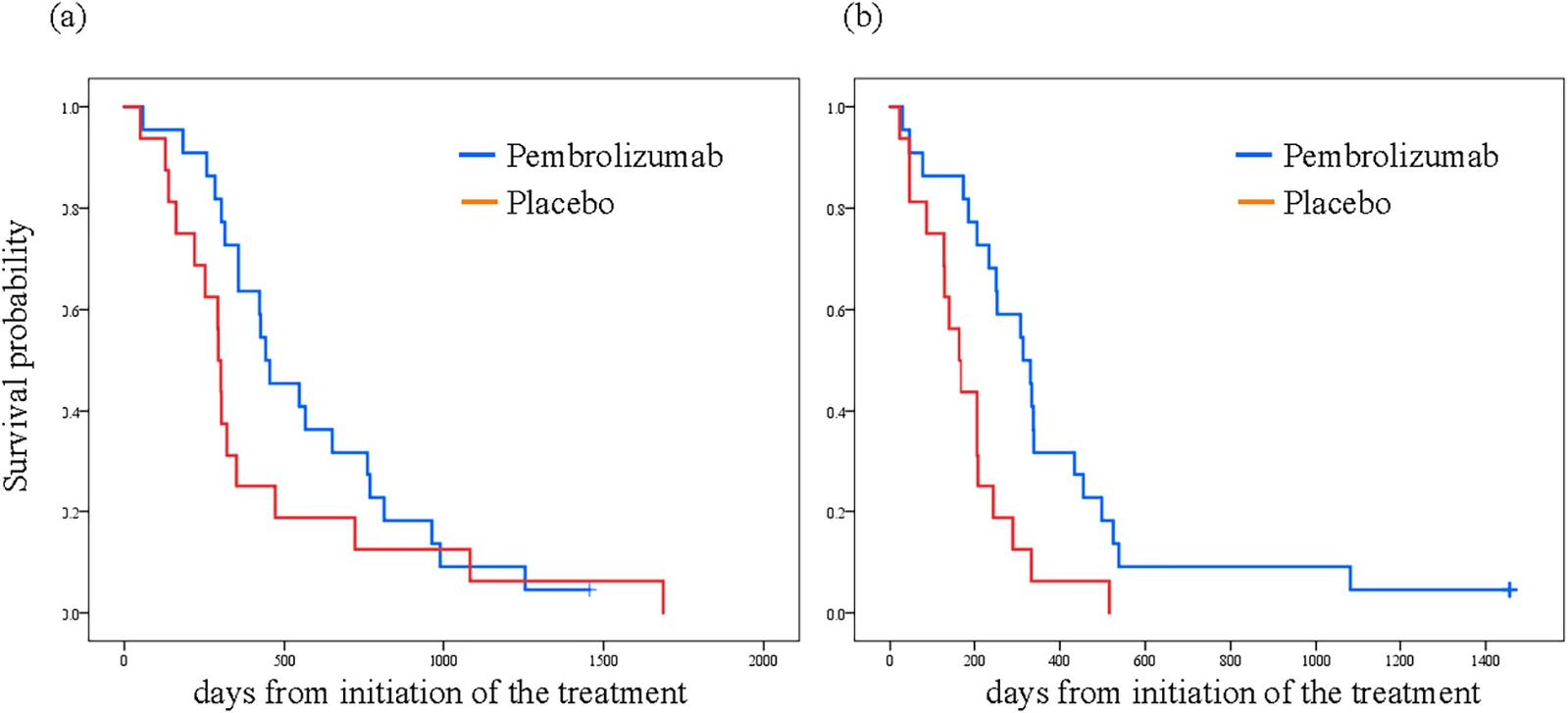
Kaplan–Meier curve of **(A)** overall survival and **(B)** progression-free survival in the actual cohort. The blue and orange lines represent the Pembrolizumab and Placebo groups, respectively.

**Table 2:** Objective response.

N (%)	Pembrolizumab+GC N=22	Placebo+GC N=16	p-value
Complete response	1 (4.5)	0	
Partial response	6 (27.2)	1 (6.3)	
Stable disease	11 (50.0)	11 (68.8)	
Progressive disease	4 (18.2)	4 (25.0)	
Objective response	7 (31.8)	1 (6.3)	0.062
Disease control	18 (81.8)	12 (75.0)	0.698

GC, gemcitabine + cisplatin.

### 3.5 Multivariable and IPW analyses

Cox regression analysis revealed that the treatment arm was an independent prognostic factor for PFS, but not a statistically significant prognostic factor for OS. The hazard ratio for PFS in the Pembrolizumab group compared with that in the Placebo group was 0.428 (95% CI, 0.180–1.0144) and that for OS was 0.707 (95% CI, 0.321–1.554) (**Tables 3A**, **B**).

**Table 3A:** Multivariable analysis for progression-free survival.

Factors	Hazard ratio	95% CI	p-value
Performance status = 0 (vs. 1)	0.436	0.183–1.040	0.061
ln(CA 19-9), restricted cubic spline^†^	–	–	0.057^‡^
Locally advanced disease (vs. Metastatic)	0.733	0.326–1.648	0.453
Pembrolizumab+GC (vs. placebo+GC)	0.428	0.180–1.014	0.054

CI, confidence interval; CA 19-9, Carbohydrate antigen 19-9; GC, gemcitabine plus cisplatin.

† ln(CA 19-9) was modeled as a continuous variable using a restricted cubic spline with three knots placed at the 10th, 50th, and 90th percentiles (6.14, 217.05, and 23,648.45 U/mL, respectively).

‡ The p-value represents the overall association between CA 19–9 and overall survival. The p-value for non-linearity was 0.017.

**Table 3B:** Multivariable analysis for overall survival.

Factors	Hazard ratio	95% CI	p-value
Performance status = 0 (vs. 1)	0.677	0.294–1.558	0.359
ln(CA 19-9), restricted cubic spline^†^	–	–	0.003^‡^
Locally advanced disease (vs. Metastatic)	0.586	0.266–1.291	0.185
Pembrolizumab+GC (vs. Placebo+GC)	0.707	0.321–1.554	0.388

CI, confidence interval; CA 19-9, Carbohydrate antigen 19-9; GC, gemcitabine plus cisplatin.

† ln(CA 19-9) was modeled as a continuous variable using a restricted cubic spline with three knots placed at the 10th, 50th, and 90th percentiles (6.14, 217.05, and 23,648.45 U/mL, respectively).

‡ The p-value represents the overall association between CA 19–9 and overall survival. The p-value for non-linearity was 0.0009.

Patient backgrounds after IPW are shown in **Supplementary Table 3**, and the distribution of SMD before and after IPW is shown in **Supplementary Figure 1**. Although most covariates were reasonably balanced after IPW, residual imbalance remained for Albumin, ln(CA19-9 + 1), ECOG PS, disease status, and site of origin with SMD >0.1. In the IPW analysis, pembrolizumab was associated with a numerically lower risk of death than placebo, although the difference was not statistically significant (HR, 0.67; 95% CI, 0.29–1.56; P = 0.353). The hazard ratio for OS in the pembrolizumab group compared with that in the placebo group in the IPW cohort was HR 0.670 (95% CI, 0.288–1.559; p=0.353), and that for PFS was 0.416 (95% CI, 0.178–0.970; p=0.042). The median OSs were 14.5 (95% CI, 11.7–24.9) months and 10.0 (95% CI, 9.6–23.7) months in the pembrolizumab and placebo groups, respectively (**Figure 2A**). The median PFSs were 10.3 (95% CI, 6.8–14.3) and 6.8 (95% CI, 4.1–8.0) months in the pembrolizumab and placebo groups, respectively (**Figure 2B**).

**Figure 2:**
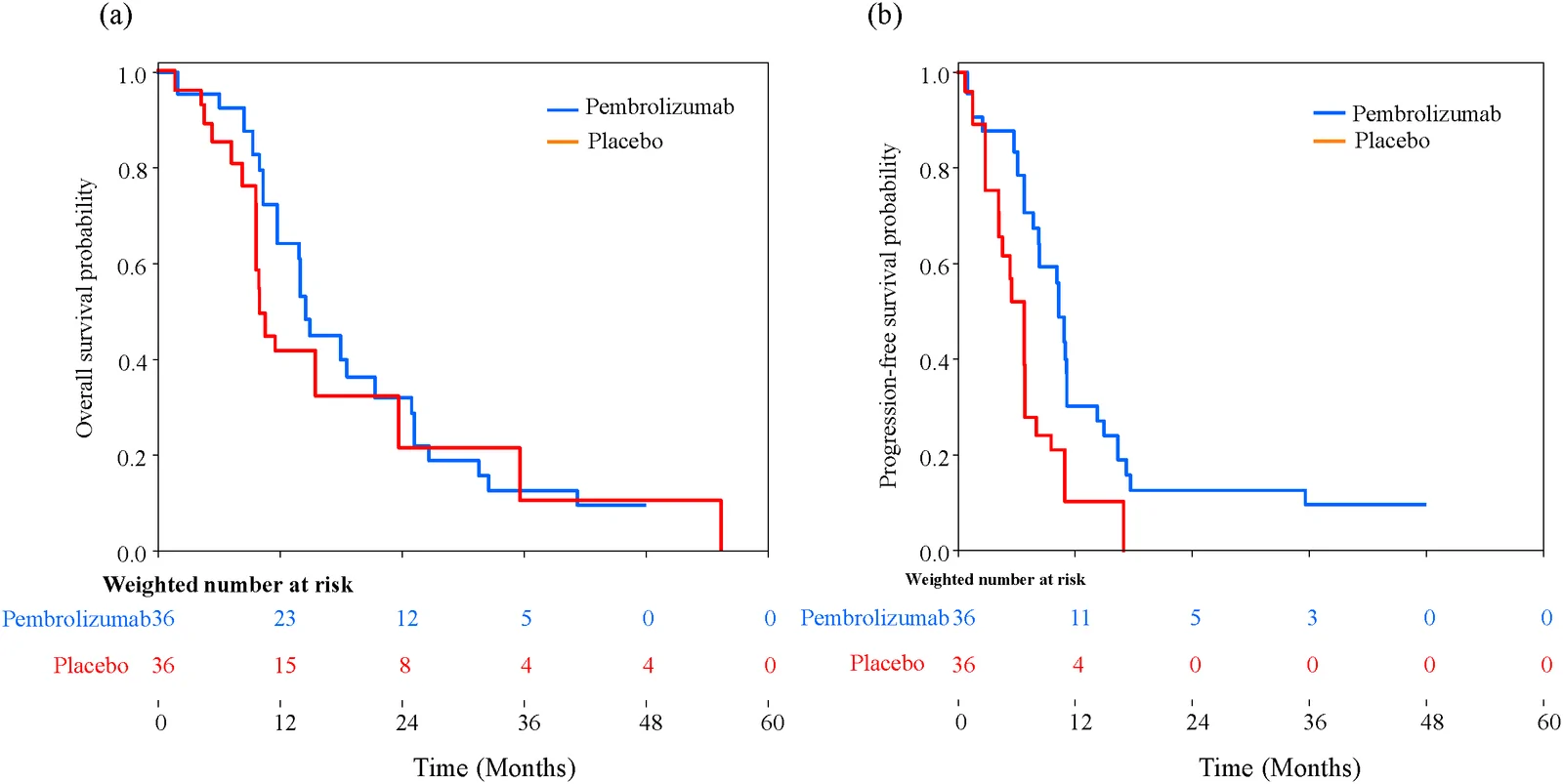
Kaplan–Meier curve of **(A)** overall and **(B)** progression-free survival in the inverse probability-weighted cohort. The blue and orange lines represent the Pembrolizumab and Placebo groups, respectively.

### 3.6 Exploratory exposure analysis

We analyzed the effect of antibiotic use before or during treatment with pembrolizumab/placebo plus Gem+CDDP on their PFS. There was no difference in PFS between patients who had history of receiving antibiotics before registration and those who had not in the pembrolizumab and placebo group with p = 0.785 and 0.848, respectively (**Supplementary Figures 2A, B**). Similarly, compared patients who received antibiotics during the anti-cancer treatment and those who did not, there was no difference in PFS of the pembrolizumab and placebo group, with p = 0.620 and 0.678, respectively (**Supplementary Figures 2C, D**). Cox hazard regression model showed treatment interaction in neither OS nor PFS by antibiotic use between the pembrolizumab and placebo group, with p = 0.972 and 0.95, respectively.

## 4 Discussion

We conducted a *post-hoc* exploratory analysis of 38 patients from our center who were enrolled in the KEYNOTE-966 trial with institutional experience for considering a supportive-care strategy for aBTC during the ICI era. The treatment effect estimates showed significantly better PFS and numerically better OS in the pembrolizumab group than in the placebo group, which were similar trends as the KEYNOTE-966 entire cohort. Of note, the present study was conducted at a Japanese institution and therefore may reflect population and clinical-practice characteristics specific to Japan. Furthermore, the observed treatment effect of pembrolizumab may have been affected by some confounding. Additionally, the favorable treatment effect observed in our institutional subgroup should not be interpreted as evidence that avoidance of dexamethasone or antibiotics enhances the efficacy of pembrolizumab. The present findings should instead be regarded as hypothesis-generating observations that may motivate prospective evaluation of supportive-care strategies during the treatment using ICIs in patients with aBTC.

In a meta-analysis of lung, genitourinary, skin, gastrointestinal, gynecological, and head/neck tumors, antibiotic use before or after the initiation of antitumor treatments with ICI was reported to negatively affect the antitumor effect (8). In contrast, the efficacy of pembrolizumab in combination with Gem+CDDP was consistent in patients who had used antibiotics within 1 month of treatment initiation in KEYNOTE-966 though only approximately 10% of the patients in the entire KEYNOTE-966 cohort had biliary stent placement and a history of antibiotic use within 1 month of the start of the study, which suggests that most of the patients did not receive antibiotics during the study treatment (4). The TOPAZ-1 trial also showed that hazard ratios of OS and PFS were approximately 0.75, even in patients who used antibiotics before or after the initiation of treatment (14). Our findings do not establish that antibiotic avoidance improves the efficacy of pembrolizumab, and we think the negative effects of antibiotics on the treatment efficacy of ICIs are minimal in advanced BTCs. Among the microbiomes, *Ruminococcaceae* (15), *Bifidobacterium* (16), and *Enterococcus* species (17) have been reported to promote the response to ICIs. In contrast, *Bacteroides* (18), *Fusovacterium* (19) and *Clostridium* species (6, 20) have been reported to associate with impaired antitumor immunity and resistance to ICIs. Among these, *Bacteroides* (21) *and Fusobacterium* species have been reported to promote the incidence of BTC, and patients with BTC would tend to have such species predominantly in their gut microbiomes at the time of BTC onset. Consequently, we speculate that ICIs had no substantial impact on the survival of patients with BTCs as much as on those with the other type of cancer, and that there was no difference in the efficacy of ICIs between patients who used antibiotics and those who did not. From another perspective, comparing the efficacy of ICIs between patients who received antibiotics and those who did not would be unfair. The disease extent and stage would be different between patients who required antibiotics and those who did not, because patients with advanced BTC who required antibiotics would have biliary stricture with biliary stents, whereas those not requiring it would not have biliary stricture. To evaluate the true impact of antibiotics on the efficacy of ICIs in patients with advanced BTCs, the outcomes should be compared between patients who received antibiotics as needed and those who did not, even when they suffered an infectious disease that could not be realized. We tried to avoid using antibiotics in case of cholangitis by managing it with biliary drainage alone; however, despite the median duration of administration being only 4 days, which was shorter than that recommended by Tokyo guidelines 2018 (22), 11 out of 13 (85%) patients who experienced cholangitis received antibiotics. Finally, given the potential risk of undertreating cholangitis, antibiotics should not be withheld solely to preserve ICI efficacy in patients with advanced BTC.

One study has concluded that corticosteroids do not have a negative impact on ICI efficacy (23); however, these included patients who received corticosteroids for the treatment of irAEs. Patients who developed irAEs were reported to have a good prognosis (24, 25), and there may be confounding factors between corticosteroid use and prognosis. Some reports have shown that prednisolone at a dose of 10 mg/day or less does not worsen the prognosis of patients treated with ICIs, even when it was used from the initiation of ICIs (12). DEX at a dose of 9.9 mg/day on day 1 is equivalent to 50 mg/day (26), and has a longer half-life time than prednisolone (27). Additionally, DEX suppresses antigen-presenting cells, helper T cells, and cytotoxic T cells more than prednisolone (28). These data indicate that DEX, as an antiemetic, negatively impacts the efficacy of ICIs. Based on these data, we believe that the use of DEX should be avoided as much as possible, and 5-HT_3_ RA (29, 30) and Neurokinin-1 receptor antagonists (31–34), which have been proposed as new antiemetic treatments, should be used instead (9, 35). Although compared with that observed in the entire KEYNOTE-966 cohort (4), we detected no significant increase in the incidence of chemotherapy-induced nausea and vomiting (CINV) in the cohort assessed in the present study, the risk factors associated with CINV, such as a younger age (<60 years), history of morning sickness (36) and being female (37), should be considered. A further concern is the cost of aprepitant/fosnetupitant, which is more expensive than DEX (approximately 15–76 $/week), and should accordingly be taken into account. However, the cost burden was not so high that it would be sufficiently covered to improve survival. Additionally, avoiding DEX administration may be beneficial for glycemic control in patients with Diabetes Mellitus and insomnia.

Another aspect of potential risk of avoiding prophylactic DEX use is the incidence of irAEs. Potential irAEs and the use of systemic corticosteroid treatment for irAEs in the pembrolizumab group at our institution were more frequent than those in the KEYNOTE-966 trial. This suggests that antiemetic prophylactic DEX may decrease the incidence and the severity of irAEs.

Our study has some limitations. First, this institutional subgroup analysis was not pre-specified, and the number of patients included was too small to conclude the efficacy of pembrolizumab plus Gem+CDDP compared with placebo plus Gem+CDDP in our cohort. There were some imbalances between the two treatment groups, such as disease stage, serum level of CRP and CA 19-9. We conducted multivariate analysis and IPW analysis to consider the differences in patient’s background though these analyses could not eliminate the possibility of confounding factors. Small event numbers also limit multivariable adjustment. Second, whether our supportive care approach to minimize antibiotics and antiemetic DEX would have a survival benefit because the institutional supportive-care strategy was applied to both randomized treatment groups. Furthermore, because of differences in sample size, patient characteristics, and study setting, the magnitude of adding pembrolizumab on Gem+CDDP in our cohort cannot be directly compared with those of KEYNOTE-966 entire cohort. Therefore, our study design does not allow us to isolate the effect of dexamethasone or antibiotic avoidance on the efficacy of pembrolizumab. Third, the effect of steroids on the antitumoral activity of ICIs would vary depending on the treatment protocol because ICIs added to cytotoxic agents in other types of cancers, including melanoma, lung cancer, and gastric cancer, used a 3- or 4-week regimen, which means that the antiemetic DEX was administered only for 1–3 days within a 21- or 28-day cycle (38).

In conclusion, the findings of this institutional subgroup study indicate that the efficacy of treatment using a combination of pembrolizumab and Gem+CDDP is maintained compared with placebo and generate hypothesis that avoiding DEX use as antiemetics pronounce the efficacy of ICIs on aBTCg. A randomized controlled trial is warranted to verify the hypothesis.

## Data Availability

The original contributions presented in the study are included in the article/**Supplementary Material**. Further inquiries can be directed to the corresponding author.

## Supplementary material

Supplementary Figure 1 Standard mean differences in patient characteristics before and after the inverse probability weighting. Absolute standardized mean differences (SMDs) are shown for each covariate before and after inverse probability weighting (IPW). For CA 19-9, SMD was calculated using ln(CA 19-9 + 1) because of its markedly right-skewed distribution. The dashed and dotted vertical lines indicate absolute SMD of 0.1 and 0.2, respectively.

Supplementary Figure 2 Kaplan-Meier curves of progression-free survival in patients with history of antibiotics use before the registration **(A)** in the pembrolizumab group and **(B)** in the placebo group, and that in patients received antibiotics after the registration **(C)** in the pembrolizumab group and **(D)** in the placebo group.

Supplementary Figure 3 Adjusted restricted cubic spline for CA19–9 and **(A)** progression-free survival and **(B)** overall survival. Reference (217.05 U/mL) was the median value of the entire cohort in this study.
