# Supplementary material for: Clinical outcomes of pembrolizumab plus gemcitabine and cisplatin in a Japanese institutional subgroup of KEYNOTE-966: a dexamethasone-minimizing supportive-care approach
Source: Front Oncol. 2026 Sep 21;16:1806667. doi: 10.3389/fonc.2026.1806667 (PMC13634956; doi:10.3389/fonc.2026.1806667)
Supplement: Supplementary file 1 [file DataSheet1.docx]

Supplementary Table 1. Supportive-care for infectious and emetic events

|  | N (%) | | | |
| --- | --- | --- | --- | --- |
|  | Pembrolizumab group  N=22 | | Placebo group  N=16 | |
| Cholangitis | 9 (41) | | 4 (25) | |
| Antibiotic use | 7 (32) | | 4 (25) | |
| Biliary drainage |  | |  | |
| Pneumonitis | 1 (5) | | 0 | |
| Antibiotic use | 1 (5) | | 0 | |
|  | Emetic events, N (%) | | | |
|  | Any grades | Grade ≥3 | Any grades | Grade ≥3 |
| Nausea | 9 (41) | 0 (0) | 7 (44) | 0 (0) |
| Vomiting | 3 (14) | 0 (0) | 2 (13) | 0 (0) |
| Anorexia | 5 (23) | 1 (5) | 6 (38) | 0 (0) |
| Steroid use | 0 (0) | | 0 (0) | |
| Domperidone or Metoclopramide | 7 (32) | | 7 (44) | |

Supplementary Table 2. Incidence of immune-related adverse events

|  | Pembrolizumab group  N=22 | | Placebo group  N=16 | |
| --- | --- | --- | --- | --- |
| N (%) | Any grades | Systemic corticosteroid | Any grades | Systemic corticosteroid |
| Any events | 6 (27) | 0 | 1 (6) | 1 (6) |
| Interstitial lung disease | 4 (18) | 4 (18) | 0 | 0 |
| Thrombocytopenia | 1 (5) | 0 | 0 | 0 |
| Nausea | 1 (5) | 0 | 0 | 0 |
| Liver dysfunction | 0 | 0 | 1 (6) | 1 (6) |

Supplementary Table 3. Patient background in the inverse probability weighting propensity score analysis

|  | Pembrolizumab+GC  N=22 | Placebo+GC  N=16 | SMD |
| --- | --- | --- | --- |
| Age (year)  mean (SD) | 67.9 (7.75) | 68.0 (10.5) | 0.047 |
| Sex: Male | 55.6% | 57.8% | 0.015 |
| ECOG PS: 0 | 75.6% | 70.2% | 0.145 |
| Site of origin  Gallbladder | 24.6% | 26.5% | 0.120 |
| Disease status  Locally advanced | 34.0% | 30.0% | 0.142 |
| CRP (mg/dL)  Mean (SD) | 1.17 (1.59) | 1.15 (1.03) | 0.004 |
| Albumin (g/dL)  Mean (SD) | 3.8 (0.43) | 3.7 (0.35) | 0.188 |
| NLR  Mean (SD) | 3.3 (1.8) | 3.4 (2.4) | 0.018 |
| CA 19-9 (U/mL)  Mean (SD) | 15746.2 (59961.0) | 17199.7 (68075.3) | 0.0181^†^ |

GC, gemcitabine + cisplatin; SMD, standardized mean difference; SD, standard deviation; CRP, C-reactive protein; CA 19-9, carbohydrate antigen 19-9

^†^For CA 19-9, the standardized mean difference was calculated using ln(CA 19-9 + 1) because of its markedly right-skewed distribution.
